# Appointments to the Philadelphia Hospital

**Published:** 1860-09

**Authors:** 


APPOINTMENTS TO THE PHILADELPHIA HOSPITAL.—At a meeting of the Board of Guardians, held on the thirtieth of July, the following appointments were made:—

*Physicians.*—Drs. J. Da Costa, J. L. Ludlow, C. P. Tutt, and O. A. Judson.

*Surgeons.*—Drs. S. D. Gross, D. Hayes Agnew, R. J. Levis, and R. S. Kenderdine.

*Accoucheurs.*—Drs. R. A. F. Penrose, L. D. Harlow, W. D. Stroud, and G. J. Ziegler.

At a meeting, held July sixteenth, Dr. S. W. Butler was re-elected Superintendent of the Insane Department.

